# An Enquiry into the Physiological and Medicinal Properties of the Aconitum Napellus, &c.

**Published:** 1846-01

**Authors:** 


An Inquiry into the Physiological and Medicinal Properties of the Aconitum Napellus; to which are added, Observations on several other Species of Aconitum. By *Alexander Fleming*, M.D., President of the Royal Medical Society of Edinburgh. Octavo, pp. 160. Churchill. London, 1845.

We hail with much pleasure and satisfaction all works having for their object the elucidation of the peculiar and distinctive effects of the very different remedial agents composing the important group commonly denominated Narcotics. For there is not, we apprehend, any other physiological class of medicines which presents to the physician so many points of interest as this one, whether studied in reference to physiology, toxicology, or practical medicine.

It is well known that these agents affect, though in an unequal and different manner, the various functions of the cerebral and spinal systems; and we know no more interesting and legitimate topic of physiological research, than the investigation into the precise seat of the operation of these agents, with the view of determining the particular functions of the several portions of the nervous system.

To the toxicologist the study of the operation of narcotics presents more immediate and practical advantages. It enables him to distinguish the different kinds of narcotic poisoning, to administer the antidote proper for each case, and frequently to detect crime.

Nor is the utility of a precise knowledge of the effects of narcotics less immediate and obvious to the practical physician. A more complete insight into the nature of their action would enable him to use them with more confidence and success; and to select the particular agent adapted for each case. We are afraid that, at the present time, a large number of practitioners regard the different narcotics rather as agents possessing different degrees of activity, than as substances differing specifically from each other in the nature of their operation. To such, Dr. Fleming's work is calculated to be of great use.

Dr. Fleming's work is divided into six sections, to which he has added an Appendix. We propose to examine successively the subjects of which he treats in each section.

*Section First.*—In this section the author notices the *History, Botany,* and *Physical Characters* of *Aconitum Napellus.*

Decandolle, in his *Prodromus,* has admitted 107 kinds of *Aconitum.* These he has arranged in 22 species. But a more recent writer, Mr. G. Don, in his *Gardener's Dictionary,* has described above 160 sorts, which he has arranged in 76 species. So that very considerable uncertainty exists as to the actual number of distinct species known at the present time.

The common Aconite of the gardens of this country is *Aconitum Napellus.* Of this species Decandolle admits 29, Mr. G. Don only about 11, varieties. So that a similar uncertainty exists with respect to the limits and varieties of this species, as we have above stated to exist with regard to the genus.

Dr. Fleming follows Decandolle, and does not even allude to the differences and uncertainties with respect to the limits of the genus and species which we have here noticed. He agrees with Dr. Pereira in recommending the Aconitum Napellus as the officinal species; and, in the sixth section of his work, gives what he considers "conclusive reasons" for this preference.

Dr. Fleming does not appear to have made any experiments to determine the comparative activity of the different varieties of Aconitum Napellus cultivated in gardens in this country; at least none are mentioned in the present work. We much regret this omission.

He has made several experiments to determine the comparative activity of the different parts of the plant; and concludes that the *root* is the most eligible part for medicinal use, both on account of its greater activity, and also from the ease with which it may be obtained in large quantity. In this respect he agrees with Dr. Turnbull. The other parts of the plant are inferior in power. The *seeds* rank second, the *leaves* third, the *flowers* fourth, and the *fruit* and *stem* last in the order of medicinal activity.

The *root* is formed of two parts—a tapering root-stock, and one or more pyriform tubers attached by narrow necks to its upper part. The tuber of this year becomes the root-stock of the next, in the autumn of which it rots and dies; so that the root is biennial. The best time for gathering it is soon after the period of flowering, when the tuber possesses more activity than at any other time. It follows from Dr. Fleming's observations, that the tuber, and not the rootlets, should be selected for medicinal use. The author does not, however, directly state so; but we infer this from his remarks. We believe that the dried root sold in the London herb-shops is the second year's growth; and that the root-stalk constitutes its principal part. We know that it yields a very potent tincture.

*Second Section.*—In this section the author describes the *Physiological Action* of *Aconitum Napellus* on *Animals* [and *Vegetables*.]

In the Appendix, placed at the end of the work, the author details the individual experiments which he has made; and we there learn that the animals experimented on were the dog, rabbit, kitten, pigeon, earthworm, and infusoria.

The general effects on the "lower" animals were, according to Dr. Fleming, as follows:

"Aconite, when introduced into the system of one of the lower animals, produces, in the first instance, weakness of the limbs and staggering. The breathing then becomes either slightly accelerated, or slow and labouring. The paralysis increasing, the animal is at last unable longer to support itself, and lies down upon its side, with the extremities stretched out in a relaxed state. The general sensibility of the surface is impaired, and, towards the fatal termination, is altogether lost. Blindness, to a greater or less extent, soon supervenes: the breathing becomes gradually slower and more imperfect; and, after a few spasmodic twitches, death by *Asphyxia* ensues.

"On examination of the body immediately after death, the heart is found beating with considerable strength, nor does its action cease for some time. The peristaltic motion of the intestines also continues. The irritability of the voluntary muscles is impaired, as is evinced by their being less easily excited to contraction by mechanical irritation, than is usually the case, although they still respond readily to galvanism. General venous congestion exists; the right side of the heart is distended; there is engorgement of the venæ cavæ, of their tributary veins, and frequently of the brain; venous blood may usually be detected in the left side of the heart and in the aorta. The blood coagulates, and the muscles become rigid as usual." P. 11.

If, by the phrase "lower animals," the author means to include the dog, we deny the completeness of his history of the symptoms; which would as well apply to other narcotic poisons as to Aconite. We found our objection to his description, as well on Dr. Fleming's experiments, as on our own personal observation of the effects of this poison on animals.

He omits to mention an almost universal symptom in dogs, viz. vomiting. In the *Appendix*, the author details four experiments on dogs, illustrative of the action of the poison when introduced into the stomach; and in every one of these vomiting took place. Hence, therefore, according to his own observations, the author ought to have mentioned vomiting among the general symptoms of poisoning by Aconite.

Most of his experiments were made on the rabbit, an animal which, like the horse, cannot vomit; and, therefore, the absence of this symptom in this animal is readily accounted for.

That the vomiting, which Aconite gives rise to in the dog, does not depend on the local action of the poison on the stomach, we have ourselves proved; for this symptom occurs when the poison is introduced into the cellular tissue of the back, or leg, other parts, provided that the death is not instantaneous, as when the poison is thrown into the veins.

The omission of vomiting, from the general catalogue of the effects produced by Aconite on animals, is the more to be regretted, because it is a symptom of almost universal occurrence in poisoning by this substance in the human subject; and the value of toxicological experiments on the lower animals is principally derived from the light they are calculated to throw on the effects of poisons on man.

Another objection, which we have to make to the author's description of the effects of Aconite on animals, has reference to the order in which muscular paralysis and impaired sensibility occur. Dr. Fleming states that the muscular paralysis is one of the most characteristic symptoms of the action of the poison; and afterwards, in speaking of the effect of the poison on the common sensibility, he observes that, "it is sometimes difficult to judge of the impaired sensibility, as from the paralysis the animal has little or no power to express sensation." We admit the accuracy of this description, as applied to the rabbit (on which animal most of Dr. Fleming's experiments were made), but we deny its general application to the dog, on which animal, in most cases, the impaired sensibility may usually be recognised, prior to the occurrence of paralysis; and this is a fact of the more importance, because the same is observed in man.

In proof of our position we quote the following experiment from Dr. Pereira's *Materia Medica* :

"March 31, 1837, London Hospital. Present Mr. Adams and several medical students. A small portion of alcoholic extract of Aconite was introduced into the peritoneal sac of a strong dog, who had been kept fasting for some hours.

In a few minutes he was evidently affected. He was less capable of supporting himself, and leaned against a wall. In ten minutes was insensible to the pain caused by the introduction of pins into his legs, paws, body, tail, nose, &c. His sight, however, was unaffected; at least he winked as usual when attempts to strike him were feigned. Was not paralytic, for he walked, though not firmly. He recognised several individuals, and wagged his tail when spoken to. He made violent attempts to vomit. He then laid down, became apparently weaker, and died without a single convulsion. At one period, the action of the heart was slower than usual, and the first and second sounds of the heart were unusually clear and distinct. Subsequently, the circulation was quickened. Respiration was not disordered; nor were the bowels affected."

The statements of Dr. Fleming with respect to the action of Aconite on the muscular system and common sensibility of the lower animals, if not absolutely incorrect, are certainly calculated to lead persons unacquainted with the subject into error. Thus he says, that "one of the most characteristic symptoms of the action of the poison is muscular paralysis;" while, in speaking of the common sensibility, he merely observes that this is always more or less impaired. Now, by the word *characteristic* we understand that which marks the peculiar distinctive qualities of a thing, and in this sense muscular paralysis is not the characteristic symptom of poisoning by Aconite, since it is equally the effect of conia, and some other poisons. According to our notions, the peculiar diminution of sensibility is the characteristic symptom of poisoning by Aconite, for at present we know no other poison which produces this effect. We admit that the muscular paralysis is the general or the almost universal effect, but deny that it is the characteristic one.

At page 11, Dr. Fleming observes, that—

"In general, the *pupil* is more or less *contracted*, dilating to its natural size immediately on the cessation of the respiration. This symptom seems attributable to the same pathological cause [*congestion of the brain*] as the convulsions, and for the same reasons. It was present in all the cases in which convulsions occurred. In the two experiments, on the other hand, in which the Muriate of Aconitina was injected into the veins, and where, from the rapidity of the fatal result, very slight venous congestion could have existed, the pupils dilated, and continued to do so up to the moment of death: apparently shewing, that *dilatation* is the specific or direct effect of Aconite on the pupil."

We confess we are at a loss to understand Dr. Fleming's conclusion, "that *dilatation* is the specific or direct effect of Aconite on the pupil," when he admits that, "in general, the pupil is more or less *contracted*" after the introduction of Aconite into the system.

His lame attempt to explain away the contraction of the pupil by ascribing it to congestion of the brain, totally fails in accounting for the contraction of the pupil produced by painting the conjunctiva with a minute portion of the unguentum aconitinæ. Moreover, what reliance can be placed on the observations made in the experiments 20 and 21, in the first of which the dog died in eight or nine seconds, in the latter, in twenty-three seconds? He states that, "immediately on the cessation of the respiration [that is, as we should say, on the death of the animal], the pupil dilates to its natural size:" now, in the 20th experiment, the animal died *within* eight or nine seconds [the author guesses at the period, for the time-keeper neglected his duty in this experiment], and, in the 21st experiment, the dilatation did not occur until the expiration of fifteen seconds, and in eight seconds more, no sign of animal life was discoverable.

"The rapidity and intensity of the remote effects of Aconite, are in direct proportion to the absorbing powers of the part to which it is applied, producing no effect when placed in contact with the skin, and acting with less energy when taken into the stomach than when introduced into a serous cavity, or into the cellular tissue. It acts with the greatest energy and rapidity, when introduced directly into the circulation." P. 17.

The author is, we think, quite accurate in his remark, that "there is no important difference in character between the action of Monkshood and that of its alkaloid, Aconitina;" but he is somewhat premature in his inference that "the latter is, therefore, the true active principle of the plant." How does he explain the observations of Geiger, "that fresh Aconite yields by distillation an acrid liquor having an unpleasant odour and an irritant effect on the eyes?" For all that Dr. Fleming has advanced, Aconite may have two active principles of unequal volatility, but possessed of similar powers, just as cinchona bark has two alkaloids.

*Section Third.*—In this section the author discusses the *Physiological Action of the Aconitum Napellus on Man.*

In describing the topical action of Monkshood, he observes that this plant, when applied externally, acts as a *direct sedative to the nerves of sensation.* This is quite true as far as it goes; but it scarcely goes far enough; for, besides being sedative, Aconite is alterative also, since the topical effects are not those which would arise solely from sedation. The heat, the peculiar tingling, the sense of swelling and distention, fully prove that sensation or feeling is altered in quality as well as lessened in intensity.

The author makes a very curious and interesting observation, which is new to us. After stating that *contraction of the pupil* speedily takes place and continues for several hours in consequence of painting the conjunctiva with the ointment of Aconitina, and referring to Dr. Pereira's experiments in some amaurotic cases, the author observes :—

"When, on the other hand, the ointment of the alkaloid, or the tincture of the root is applied to the temple or forehead, the pupil occasionally becomes *dilated.* I have only seen this latter occurrence in two cases, in both of which it was accompanied by partial blindness of the same eye. These phenomena are either effected by reflex action through the fifth and third nerves, or perhaps by imbibition; but why such entirely opposite effects should ensue in the two cases, it is difficult to understand.

"I may mention that I have several times anointed the eyelids with the alcoholic extract in the same way as Belladonna is usually applied for the purpose of producing dilatation of the pupil, but without the slightest effect; a circumstance perhaps accounted for by the fact that the action of Aconite on the skin is only developed after considerable friction has been employed." P. 22.

The second division of this section is headed "*Physiological Action on Man in small or Medicinal Doses;*" but this is a misnomer, since it includes the effects of poisonous and fatal doses. Surely the author would not denominate a quantity of Monkshood sufficient to destroy life as a "*medicinal*" dose?

In taking a general view of the ordinary effects of Aconite, the author considers them under four degrees of operation.

"*First Degree of Operation.*—In the course of twenty minutes or half an hour, after the exhibition of five minims of the tincture, a feeling of warmth in the stomach is usually experienced, which is occasionally accompanied by slight nausea and oppression of the breathing. After the lapse of thirty or forty minutes, this sense of warmth is diffused throughout the body, and, in a few minutes more, is attended by numbness, tingling, and a sense of distention of the lips and tongue. There is also tingling at the tips of the fingers, and a peculiar sensation is felt at the roots of the teeth. The feeling of warmth soon disappears, but the numbness and tingling of the lips and fingers continue for a period varying from one to three hours. Slight muscular weakness is generally experienced, with indisposition for exertion either mental or corporeal. In about half an hour more, the pulse is found to be diminished in strength: and in another hour, both the pulse and the respiration have become less frequent. Thus, a pulse which, in the normal state, beats seventy-two in the minute, will, by that time, have fallen to about sixty-four, and the respirations, supposing them to have been eighteen, to fifteen or sixteen.

"*Second Degree of Operation.*—Should a dose of ten minims be given at first or the first dose of five minims be succeeded in two hours by another of equal amount, these symptoms supervene more rapidly, and with greater severity. The tingling extends along the arms, and the sensibility of the surface is more or less impaired. In an hour and a half, the pulse will probably have fallen to about fifty-six beats in the minute, and become smaller and weaker than before, still maintaining, however, perfect regularity. The respirations will have diminished to about thirteen, presenting, at the same time, a slow labouring character. Great muscular debility is now experienced; and giddiness, with confusion of sight, comes on when the erect posture is assumed. The individual sinks into a lethargic condition, evinces great disinclination to be disturbed, although he rarely falls asleep, and complains much of chilliness, particularly in the extremities, which are cold to the touch. These phenomena continue in their full intensity from three to five hours, when they gradually disappear, a sensation of languor which lasts for several hours more alone remaining.

"This is the utmost extent to which I would recommend the physiological effects of Aconite to be carried, in order to obtain, with safety and success, its therapeutic action.

"*Third Degree of Operation.*—On the administration of five minims more, two hours subsequent to the last dose, the sense of warmth and the numbness and tingling, again spread rapidly over the body. The sensibility of the surface is still farther diminished; lancinating pains in the joints are occasionally complained of; the headache, vertigo, and dimness of vision, are aggravated; the countenance grows pale and anxious; the muscular feebleness increases; the voice becomes weak, and the individual is frequently impressed with the dread of approaching dissolution. Occasionally, the pulse is reduced still further in strength and frequency, perhaps falling to 40, or even 36 beats per minute, but still maintaining its regularity. More frequently, however, it rises to 70 or 80, and becomes small, weak, and probably more or less irregular. The respiratory movements are also irregular, being either short and hurried, or deep and sighing. The surface is moist, and still farther reduced in temperature. Sickness may now come on; and, if formerly present, is much aggravated, and probably attended by vomiting. These symptoms do not entirely subside for one or two days.

"*Fourth Degree of Operation.*—If the administration be carried further, the symptoms assume a more alarming character. The countenance becomes pale and sunken; froth issues from the mouth, and the prostration increases. Two patients thus affected stated, that they felt as if dying from excessive loss of blood. Consciousness usually remains; or there may be slight wandering delirium, as occurs also after profuse hæmorrhage. The voice is whispering, or is altogether lost. The pulse becomes still smaller, weaker, and more irregular; and the breathing more imperfect. The surface is colder than before, and is covered with a clammy sweat.*

"When the action of the drug is carried to a fatal extent, the individual becomes entirely blind, deaf, and speechless. He either retains his consciousness to the last, or is affected with slight wandering delirium; the pupils are dilated; general muscular tremors, or even slight convulsions, supervene; the pulse becomes imperceptible, both at the wrist and heart; the temperature of the surface sinks still lower than before; and at length, after a few hurried gasps, death by *syncope* takes place." P. 25.

This appears to us to be the most appropriate place for referring to the recent melancholy death of Dr. Male, which was caused, according to the opinion of his medical attendant, by the depressing influence on the nervous system of accumulated doses of tincture of Aconite, taken for the relief of pains in the back and loins, in consequence of the deceased having recently read a book (Dr. Fleming's) treating upon the advantages of Aconite in similar pains.

After having carefully perused the medical evidence given at the inquest, our opinion, grounded on an extensive experience with this drug, is, that Dr. Male's death was not attributable to the Aconite which he took. His symptoms were cold extremities, coldness and clamminess of the general surface of the skin, quick (130) and feeble pulse, with cramps and pains in his legs, and spasmodic pains in his stomach. He had been also suffering from diarrhœa for a few days. He gradually sank into a torpid state, from which, however, he could be easily roused, and then his intellects were clear. There was no paralysis, nor could there have been loss of speech, since it is stated that he took an affectionate leave of his friends; and reminded his medical attendant that for thirty-five years they had lived together in uninterrupted friendship.

It is obvious, therefore, that his symptoms were those of collapse and depression, which are of frequent occurrence from various causes, as cholera, the latter stages of diarrhœa, &c. That, generally speaking, they attend an overdose of Aconite cannot be denied; but, in Dr. Male's case, the symptoms characteristic of Aconite poisoning were absent, viz. the numbness, tingling and other phenomena of disordered sensibility, and difficulty of articulation. We have observed in our use of Aconite, that numb-

---

* "The effects detailed in the above paragraph are derived from four cases in which they occurred accidentally. In one of these the symptoms supervened prematurely, a circumstance to be attributed to the peculiar idiosyncrasy of the patient; in another they were induced by an error on the part of the attendant (see Appendix, Part II. Case IV., report of December 21); and in the remaining two were brought on by the patients themselves, who, in their anxiety to obtain complete relief from severe pain, took more of the medicine than was prescribed (see Appendix, Part III., Case VIII.) The bad symptoms in all these cases were removed by appropriate remedies, and the patients recovered. The train of symptoms characterising the action of the drug when carried to a fatal extent, is derived from the published cases of poisoning, and is introduced to complete the view of its physiological effects, and to shew forcibly its peculiar depressing agency on the heart."

ness and tingling are among the earliest and most marked symptoms of the operation of this drug.

In enumerating the effects of poisonous doses of Aconite, Dr. Fleming mentions *dilatation of the pupils* as one of the symptoms. But, in turning to his Appendix of cases of poisoning, it appears, that of nine cases which he records, the condition of the pupil is not noticed in four of them ; while in two, contraction of the pupil occurred, and in three only is dilatation mentioned.

The author concludes that Aconite is a direct sedative poison, and that, according to the amount of the dose and consequent rapidity with which the fatal result ensues, there are three varieties in the symptoms and mode of death.

*First.*—It may prove fatal by a *powerfully sedative impression on the nervous system.*

*Secondly.*—It may prove fatal by *suspension of the respiratory function.*

*Thirdly.*—It may prove fatal by *syncope.*

The following is the mode of treating cases of poisoning by Aconite.

"Provided vomiting to a sufficient extent has not already been excited as an effect of the poison itself, an emetic must at once be administered. If a sufficient time have elapsed for the poison to have reached the intestinal tube, a cathartic ought then to be given, and followed up, if necessary, by purgativ injections.

"Tannic acid, from its power of forming insoluble compounds with the vegetable alkaloids, may be expected to be useful in neutralising the poison. The experiments on rabbits formerly noticed, shew that the gastric juice of these animals possesses a similar property. An infusion of the stomach of the rabbit, and probably of certain other herbivorous animals, might, therefore, be serviceable in poisoning by Aconite, although, from the length of time which it requires to act, this is more than doubtful.

"In order to combat the *remote effects* of the poison, which have been shewn to be powerfully sedative, a *stimulant* line of treatment must be rigidly enforced. Brandy and hot water, with ammonia, will be found most efficacious. Strong coffee has also been used with decided advantage. From my own observation, I am of opinion that great benefit is to be derived from friction with warm cloths and spirituous liniments, especially along the course of the spine and on the extremities. By thus stimulating the capillaries, the heart's action seems to be materially assisted. Sinapisms, or bottles of hot water, should also be applied to the præcordia and extremities.

"Should convulsions come on, the jugular vein ought to be opened, and a moderate quantity of blood withdrawn. By this means not only will the congestion of the brain be removed, but relief will be afforded to the heart, the right side of which is, in such cases, much engorged.

"Where there is much dyspnœa recourse may be had to artificial respiration, which will be of service not only in maintaining the function of the lungs, but also in contributing to keep up the action of the heart, and thus diminishing the tendency to syncope.

"When the action of the heart is becoming very feeble, the effect of slight galvanic shocks passed through it may be tried. In such cases, acupuncture of its walls has been recommended by Carraro." P. 53.

*Section Fourth.*—This section is devoted to the *therapeutic action of Aconitum Napellus.*

According to the author, the therapeutic properties, of which we may beneficially take advantage, are those of an *anodyne, anti-neuralgic, calmative, antispasmodic* and *antiphlogistic* (sedative to the circulation). Its *diuretic* and *diaphoretic* properties are, he thinks, too feeble and uncertain to be made available in practice ; and a more extensive trial of its *deobstruent* properties is advisable.

He arranges the diseases for which Aconite is available in three divisions ; the first including Neuralgia in its various forms, Cephalalgia, and the general pains of Fever ; the second, Diseases of the Heart ; and the third, Rheumatism, Erysipelas, Carcinoma, &c.

Under the head of *Neuralgia,* the author refers to the use of Aconite in Hemicrania, Tic Douloureux, Odontalgia, Otalgia, Thoracic and Intercostal Neuralgia, Spinal Irritation, Neuralgia of the Extremities, Sciatica, Angina Pectoris, and Gastralgia. With the exception of Angina Pectoris the author speaks from his own experience of the successful treatment of all these diseases by Aconite ; and in the excepted case, refers to the experience of Dr. Copland. We do not wish to speak at all disparagingly of the therapeutic powers of this really valuable remedy, but we are satisfied that the author has greatly over-rated its therapeutical value, and practical men, when they fail to obtain the successful results which Dr. Fleming appears to have met with, will, we are sadly afraid, discard the use of it, and thereby actually lose the aid of a very useful therapeutic agent.

" Pereira, Copland, Watson, Skey, and others, are of opinion that the external application of the remedy is more likely to be attended with success in neuralgia than its internal administration ; while Hufeland, Busse, and Tealier, give a decided preference to the latter. The table shews that both occasionally succeed in the worst cases. Our selection of the mode of treatment must be guided, in a great measure, by the nature and cause of the affection, as far as they can be ascertained. Should it appear to be caused by inflammation, either in the painful part or in the nerve farther up in its course, or should it be traceable to sympathetic irritation, the internal use of the remedy is more likely to be beneficial ; if, on the other hand, it seem to arise from some local irritation applied to the nerve, or is merely functional, its topical application will probably be sufficient. In every case where the method of treatment adopted fails, the other should be had recourse to. It is hardly necessary to add that, in recommending Aconite in the treatment of neuralgic disorders, I would not have it used to the neglect of that due attention to the secretions and excretions which is indispensable for the successful application of any remedy." P. 60.

We quite agree with the author as to the value of the internal as well as external use of the remedy ; but differ altogether from him as to the power of Aconite to cure neuralgia arising from inflammation. We have over and over again seen it completely fail in this case ; and our own belief is, that Aconite is mostly successful in, and in fact is chiefly adapted for, functional neuralgia, that is neuralgia unaccompanied with any obvious inflammation or other organic cause.

We think also that the author has greatly over-rated the efficacy of Aconite in Sciatica.

" Of twelve cases of *sciatica* in which I have used the Aconite, seven complete and two temporary cures were effected ; two cases were partially relieved, and in one only was no benefit experienced. An analysis of these cases will be found in the table ; and one of them is, for the sake of illustration, detailed in the Appendix. As far as my own experience goes, I believe it will be found most useful in those cases of sciatica, which appear to owe their origin to a congested or inflammatory condition of the nerve." P. 64.

Our experience of Aconite in Sciatica is, that in all the severe forms of the disease it fails to give much, and in many cases, any relief. Slight cases frequently get well under its use; but we are very much inclined to ascribe the recovery to nature rather than to art.

In speaking of the use of Aconite in *diseases of the heart*, the author observes that—

"In all those cases where the indication is clearly to diminish the action of the heart, Aconite is a most valuable remedy. In *functional derangement* it will often be found—in conjunction with appropriate treatment in respect to diet, regimen, &c.—equal to obtaining a complete cure. In *certain* cases of organic disease, its use is followed by great alleviation of the painful symptoms. There is a large class of cases, however, where sedative remedies are decidedly contra-indicated, but where, notwithstanding, they are too often recklessly administered. I refer to those conditions of the heart where, from some obstruction, that organ is unable to transmit the necessary quantity of blood by the usual number of pulsations, and is forced to make up for such inadequacy, by more frequent and forcible contractions. Here it is obvious that the effect of further reduction of its power would be such increased frequency, as would then be necessary to enable it to perform its task. In illustration of this, I may state that I have seen the administration of Aconite in such circumstances followed by augmented velocity, and a proportionate decrease of the force of the pulse. Where, however, it is really desirable to reduce the action of the heart, as in simple hypertrophy, functional disorder, &c., Aconite seems to be superior to digitalis, the remedy usually resorted to in such cases, and for the following reasons: Aconite is, from the first, a pure sedative, while the depressing effect of digitalis is alleged to be preceded by a stimulant action; and many bear testimony to the injurious effects arising from this primary excitement. Aconite acts much more uniformly than digitalis, which not unfrequently fails to produce the desired effect; while its primary stimulant action is said occasionally to continue, without being followed by depression. The former operates more rapidly, in the course of an hour or two, and its action can be maintained with safety by repeated small doses. It is usually, on the other hand, a day or two, or even longer, before the latter exerts any sedative influence on the heart, while there is always a risk attending its long-continued use, from its tendency to accumulate in the system." P. 68.

We sincerely hope that the author's experience of the efficacy of Aconite in *acute rheumatism* may be confirmed by subsequent experience.

"The annexed table, which is composed of my own cases, and all those recorded by others which I have met with, shows that the average period required to effect a cure [of acute rheumatism] under this treatment, is 5·6 days; the usual duration of the disease, under the ordinary treatment, being about a fortnight or three weeks. In three instances, a complete cure was effected in two days; in one, in three days; and in six, in four days. The lowest averages of the duration of the treatment of acute rheumatism are, as far as I know, those furnished by Drs. Hope and Corrigan, the former of whom found few cases which remained under treatment for more than a week; while the latter, who treated the disease by opium, gives nine days as the average. The improvement following the administration of Aconite is often very speedy, some alleviation of the pains being occasionally experienced in the course of an hour after the first dose has been taken, while there are few cases in which decided relief, with abatement of the redness, tension, and tenderness, is not obtained in a few hours. A longer period seems to be required to disperse the inflammation in the smaller joints than in the larger ones." P. 70.

We can bear testimony to the value of both the internal and external use of Aconite in *chronic rheumatism.*

"Aconite may be used both internally and externally in this disease. The internal administration seems to me preferable in what has been termed the active chronic rheumatism; that variety which is, perhaps, properly speaking, only a very mild form of the acute rheumatism, being attended with some heat and swelling of the part affected, and slight constitutional disturbance. On the other hand, I would recommend the external application of the tincture in what is called the passive chronic rheumatism, 'characterised rather by coldness and stiffness of the painful joints, with entire absence of constitutional fever.' In every case, however, should the mode of treatment adopted fail to afford relief, the other should be had recourse to; while it is frequently of service to combine the internal and external use of the remedy." P. 74.

*Section Fifth.*—In this section the author discusses the *administration* of Aconite, and recommends for medicinal use the *tincture* and the *alcoholic extract of the root.*

"TINCTURA ACONITI.

"Take of root of A. Napellas, carefully dried and finely powdered, sixteen ounces Troy; rectified spirit, sixteen fluid ounces; macerate for four days; then pack into percolator; add rectified spirit until twenty-four ounces of tincture are obtained.

"It is beautifully transparent, of the colour of sherry wine, and the taste is slightly bitter.

"EXTRACTUM ALCOHOLICUM ACONITI.

"This is prepared by distilling, at a low temperature, the spirit from the tincture, until the consistence of an extract has been obtained. The process should be completed in a vapour-bath.

"Its colour is dark brown, or almost black; it has an agreeable smell, and bitter taste. The dose is one-third of a grain, thrice daily, commencing with the one-sixth of a grain." P. 80.

We much regret that Dr. Fleming has thought it requisite to give a formula for a tincture of Aconite, yielding a preparation much stronger than that usually kept in the shops. Such an augmentation of strength is unnecessary and dangerous. Dr. Turnbull recommends one pound (= 5760 grs. troy) of the root to two pounds of rectified spirit (=11520 grs. troy), or one part root to two parts spirit by weight. Dr. Pereira estimates the spirit by measure, and directs one pound of root (= 5760 grs. troy) to one and a half imperial pints (= 10998 grs. troy), or one part root to $1\frac{9}{10}$th parts of spirit; and the tincture obtained by either of these formulæ, is strong enough for all medicinal purposes. Dr. Fleming, however, orders sixteen ounces (= 7680 grs.) of the root to 24 fluidounces (= 8799 grs.) of rectified spirit, or one part root to $1\cdot\frac{145}{1000}$ of rectified spirit by weight. The relative strengths of the three tinctures, therefore, are as follows:—

| | |
|---|---|
| Dr. Turnbull's . . . . | Root 500 to Spirit 1000. |
| Dr. Pereira's . . . . . | Root 523 to Spirit 1000. |
| Dr. Fleming's . . . . . | Root 872 to Spirit 1000. |

We shall let Dr. Fleming speak for himself as to the internal administration of the tincture.

"I prefer the tincture for internal administration, from its greater uniformity of action.

"The method of administering it varies according to the object in view.

"As an *anodyne, antineuralgic,* and *calmative,* five minims ought to be given at first, three times daily, to be increased daily to the extent of one minim each dose, until the physiological effects described under the second degree of operation have been produced.

"As an *antiphlogistic,* five minims ought to be given at first, and repeated in four hours; by which means the second degree of operation will, in all likelihood, have been induced. In order to sustain the sedative action thus developed, two and a-half minims are to be given every three or four hours, or less frequently, according to the effect produced.

"Where this mode of administration is adopted, it is *absolutely necessary* that the patient should be seen, and his pulse examined, before the exhibition of each dose. When this cannot be done, the remedy may be given in the manner pointed out for its use as an anodyne and calmative.

"The *best method* of administering the remedy in *diseases of the heart,* is to give it in smaller doses than those recommended for its use as an anodyne, but more frequently repeated, as three or four minims five times daily.

"Sickness may be avoided or checked by an effervescing draught, administered with, or immediately after, the dose." P. 82.

With regard to its external use, he justly observes, that the tincture is an excellent substitute for Aconitina.

"One or more drachms of it are to be rubbed over the affected part three times daily, the friction being continued at each time for a quarter of an hour, or, indeed, until the topical effects of the drug are fully developed.

"It is hardly necessary to add, that, when there is any abrasion of the skin, the external application of either of these preparations may be attended with danger." P. 83.

*Section Sixth.*—In this section the author treats of the *physiological action of the other species of Aconitum,* which he arranges, after Decandolle, in four sections. We subjoin his results.

### Section I.—Anthora.

1. *Aconitum Anthora.*—The root has no power of exciting numbness or tingling. Half an ounce of the tincture was without any effect whatever. One ounce occasioned warmth and sweating.

### Section II.—Lycoctonum.

2. *Aconitum Lycoctonum.*—Half an ounce of the tincture had no effect.

3. *Aconitum barbatum.*—Half an ounce of the tincture produced slight nausea.

4. *Aconitum ochroleucum.*—From ten to thirty minims of the tincture occasioned warmth and diaphoresis, but no numbness or tingling. Var. *puberulum.* Half an ounce [of the tincture] produced slight and transient warmth of the surface.

### Section III.—Cammarum.

5. *Aconitum paniculatum.*—The plants of this species, on which the observations have been made, were raised from roots sent by Decandolle.

Half an ounce of the tincture was without effect. Dr. Christison also confirms the inactivity of this species.

6. *Aconitum variegatum* } inert.

7. *Aconitum Lasiostonum* }

Section IV.—Napellus.

8. *Aconitum Napellus*.—This and all other species of this section are equally active both as regards their topical and medicinal action,

The tinctures of the roots of the above species were prepared in every case with a drachm of the root [*fresh or dried?*] to an ounce and half [*by measure or weight?*] of spirit, [*rectified or proof?*]

As the author directs the tincture of Aconitum Napellus to be prepared with rectified spirit, we presume that he used spirit of the same strength in making experiments on the other species. If not, his comparative observations are of little value.

Assuming, however, that he did, we cannot reconcile his statements of the effects of these tinctures with the known effects of alcohol; for Dr. Fleming found that half an ounce of several of these tinctures was without any effect whatever in females, and one ounce only occasioned warmth and sweating. Were his patients whiskey-drinkers? The gin usually sold to the spirit-shops in this metropolis is 22 per cent. *under proof*, and as retailed is in general still farther diluted. Yet Dr. Fleming's patients took a fluidounce of rectified spirit 56 or 58 per cent. *over proof*, without perceiving any effect beyond warmth and sweating!

Dr. Fleming's experience of the inactivity of several species of Aconitum are very interesting; but his statements must, when in opposition to the observations of previous writers, be received with considerable caution. It is well known, that Dr. Christison has recently found that the *Oenanthe crocata*, growing in the neighbourhood of Edinburgh, is quite inert, though the same species growing in the neighbourhood of London is a most virulent poison. May the same be the case with some species of Aconitum?

The London College has admitted into its Pharmacopœia *Aconitum paniculatum* Decand. as the officinal species, under the belief, we presume, that this was the species employed by Störck, Decandolle having given the name *Storkianum* to a variety of this species, and declared it to be the plant described by Störck. Dr. Fleming does not state which of the seven varieties of *A. paniculatum*, admitted by Decandolle, was the one he tried. Making due allowance for Störck's exaggeration of the therapeutical value of Aconite, it cannot be doubted, we presume, that the species he employed possessed considerable activity; and if, therefore, *A. paniculatum* be inert, we conclude this was not the species employed by Störck. However, before we arrive at this conclusion, it is necessary to know, in the first place, which of the seven varieties of *A. paniculatum*, mentioned by Decandolle, was the plant used by Dr. Fleming; and, secondly, whether *A. paniculatum* is always as inert as Dr. Fleming found it to be when growing in the Edinburgh botanic garden.

Whatever be the result of the inquiry, we feel that Dr. Fleming has done good service to Materia Medica by directing attention to the matter.

In speaking of *Aconitum ferox* Dr. Fleming states that in India " an oil is said to be distilled from it." Is not this an error?

In an *Appendix* to his work, Dr. Fleming details his experiments illustrative of the Physiological action of the *Aconitum Napellus* on animals, and gives cases illustrative of the physiological, therapeutical, and toxicological action of the same plant on man. These, however, we think it unnecessary to notice farther.

In concluding our notice of Dr. Fleming's work, we have great pleasure in recording our opinion of its utility, and in recommending it to the perusal of our readers.

